# Mismatch between Bioluminescence Imaging (BLI) and MRI When Evaluating Glioblastoma Growth: Lessons from a Study Where BLI Suggested “Regression” while MRI Showed “Progression”

**DOI:** 10.3390/cancers15061919

**Published:** 2023-03-22

**Authors:** Mathilde Bausart, Elia Bozzato, Nicolas Joudiou, Xanthippi Koutsoumpou, Bella Manshian, Véronique Préat, Bernard Gallez

**Affiliations:** 1 Advanced Drug Delivery and Biomaterials (ADDB) Research Group, Louvain Drug Research Institute, Université Catholique de Louvain (UCLouvain), 1200 Brussels, Belgium; 2 Nuclear and Electron Spin Technologies (NEST) Platform, Louvain Drug Research Institute, Université Catholique de Louvain (UCLouvain), 1200 Brussels, Belgium; 3 Department of Imaging and Pathology, Translational Cell and Tissue Research Unit, Katholiek Universiteit Leuven (KULeuven), 3000 Leuven, Belgium; 4 Biomedical Magnetic Resonance (REMA) Research Group, Louvain Drug Research Institute, Université Catholique de Louvain (UCLouvain), 1200 Brussels, Belgium

**Keywords:** glioblastoma, GL261, luciferase, bioluminescence imaging, magnetic resonance imaging, optical imaging, artifacts, reporter gene

## Abstract

**Simple Summary:**

The GL261 murine preclinical model is one of the most commonly used models to study the impact of new GBM treatments. To assess tumor growth, the use of noninvasive imaging is needed. Bioluminescence imaging (BLI) is particularly popular because of its easy access compared to magnetic resonance imaging (MRI). In our study, by comparing in vivo tumor growth of wild-type and luciferase-expressing GL261 models, we observed a difference in the estimation of tumor growth by BLI and MRI in a specific model. The divergence in results was explained by the instability in luciferase expression after transduction. Our study showed that the use of multi-modality imaging prevents possible errors in tumor growth evaluation.

**Abstract:**

Orthotopic glioblastoma xenografts are paramount for evaluating the effect of innovative anti-cancer treatments. In longitudinal studies, tumor growth (or regression) of glioblastoma can only be monitored by noninvasive imaging. For this purpose, bioluminescence imaging (BLI) has gained popularity because of its low cost and easy access. In the context of the development of new nanomedicines for treating glioblastoma, we were using luciferase-expressing GL261 cell lines. Incidentally, using BLI in a specific GL261 glioblastoma model with cells expressing both luciferase and the green fluorescent protein (GL261-luc-GFP), we observed an apparent spontaneous regression. By contrast, the magnetic resonance imaging (MRI) analysis revealed that the tumors were actually growing over time. For other models (GL261 expressing only luciferase and U87 expressing both luciferase and GFP), data from BLI and MRI correlated well. We found that the divergence in results coming from different imaging modalities was not due to the tumor localization nor the penetration depth of light but was rather linked to the instability in luciferase expression in the viral construct used for the GL261-luc-GFP model. In conclusion, the use of multi-modality imaging prevents possible errors in tumor growth evaluation, and checking the stability of luciferase expression is mandatory when using BLI as the sole imaging modality.

## 1. Introduction

Glioblastoma (GBM) is the most common and aggressive brain tumor in adults. The standard of care therapy for GBM includes surgical resection followed by radiotherapy and concomitant temozolomide chemotherapy. However, this therapy has limited success. Once GBM recurrence occurs, therapeutic options for patients are limited, and the prognosis for GBM patients is very poor [1,2,3]. Therefore, the development of innovative treatments is critically needed. Preclinical orthotopic GBM xenografts are essential for analyzing the biology of GBM, identifying new therapeutic targets, and evaluating the potential of new therapeutic strategies [4]. These models recapitulate the complexity of GBM, including the tumor microenvironment, the interactions between cells as well as the heterogeneity in blood-brain barrier (BBB) integrity and disruption. Due to the localization within the cranial cavity, the evolution of tumor growth (or regression) of GBM models can only be monitored thanks to noninvasive imaging. Noninvasive techniques make longitudinal studies possible, require fewer animals, and have greater statistical power thanks to the use of animals as their own controls [5]. Most studies on rodent models of GBM have used magnetic resonance imaging (MRI), contrast-enhanced CT [6], or bioluminescence imaging (BLI).

MRI has been utilized for screening and detecting brain tumors in rodents using the large diversity of contrast depending on T1 and T2 relaxation times, water diffusion, or contrast enhancement using paramagnetic/superparamagnetic contrast agents [7,8]. MRI provides real-time high-resolution images specially adapted to monitor subtle variations in size as well as heterogeneity in tumor response to treatments. However, the use of preclinical MRI is an expensive technology that requires sophisticated equipment and special expertise in defining imaging protocols and data processing. BLI has gained popularity because this technology is more easily accessible without the help of a trained expert and is particularly adapted to high-throughput screening of animals [9,10,11,12]. In addition, BLI images are obtained faster than MRI images. One of the most popular methods in optical imaging relies on the expression of luciferase by the xenografted cells. Luciferase expressed by the xenografted cells uses luciferin to produce visible light. The intensity of this emitted light is supposed to correlate with the size of a given xenograft, a fact that has been previously demonstrated in subcutaneous tumors [13] and in brain tumors [14,15].

In the context of research projects aiming at evaluating innovative nanomedicines for the immunotherapy of GBM, we were using the murine GL261 model. This syngeneic, immunocompetent model is one of the most widely used when studying the impact of immunotherapies and is well characterized [16]. However, during our studies, tumor growth assessment needed to be performed by MRI, which is a complicated and time-consuming technique. In vivo bioluminescence imaging has already been successfully used to assess murine gliomas [17,18]. Therefore, in order to ease the monitoring of tumors, we developed luciferase-expressing GL261 models using cells engineered to express the gene coding for firefly luciferase (luc) and/or the green fluorescent protein (GFP), namely GL261-luc and GL261-luc-GFP. After orthotopic implantation, tumor growth was monitored using BLI with supplemental MRI analysis at several time points. BLI suggested a spontaneous regression of the GL261-luc-GFP model. However, the MRI analysis revealed that GL261-luc-GFP tumors were present and growing. This unexpected observation prompted an investigation into the origin of the contradictory information provided by BLI and MRI. Correlations were built between MRI-measured tumor volumes and light radiance for each model. We found that the BBB was disrupted in the GL261 model, excluding the problem of accessibility of luciferin to the tumor cells. Because light attenuation is large for signals originating from deeper brain tissues (relative to light attenuation from surface-tissue signals), we looked into the influence of tumor localization (as assessed by MRI) on light radiance flux. We also compared in vivo measurements to ex vivo measurements carried out on brains removed from the skull. Finally, because firefly luciferase gene instability was previously reported in some cell lines [19], we looked into the stability of luciferase expression in tumor cell lines in vitro. Our results indicate that the divergence in results coming from different imaging modalities was not due to the tumor localization nor the penetration depth of light but was rather linked to the instability in luciferase expression in the viral construct used for the GL261-luc-GFP model.

## 2. Materials and Methods

### 2.1. Glioma Cell Lines and Culture

The GL261 cell line was purchased from the DSMZ (German Collection of Microorganisms and Cell Cultures GmbH, Leibniz Institute, Germany). The GL261-luc cell line was a gift from the Biomedical MRI laboratory at the KULeuven, where they used the pCHMWS-Fluc-IRES-Puro lentiviral vector for transduction. The GL261-luc-GFP and U87MG-luc-GFP cell lines were obtained by transduction using a viral vector expressing firefly luminescent and eGFP fluorescent protein (pCH-EF1a-eGFP-T2A-Luc2-Ires-puro). The transduction was carried out as previously described [18]. After transduction, cells were treated with puromycin, sorted, and single-cell cloning was performed. The combination of optical imaging (IVIS Spectrum; PerkinElmer, Waltham, MA, USA) and imaging cytometry (ImageStreamX Mark II; Merck Millipore, Seattle, WA, USA) was used to confirm successful transduction of the cells. Supplementary explanations on viral vector production can be found in the Supplementary Materials.

GL261 cell lines were grown in Dulbecco’s Modified Eagle Medium (DMEM; Gibco™, Thermo Fisher Scientific, Waltham, MA, USA) supplemented with 10% fetal bovine serum (FBS; Gibco™, Thermo Fisher Scientific, Waltham, MA, USA) and 1% penicillin/streptomycin (Gibco™, Thermo Fisher Scientific, Waltham, MA, USA). U87MG-luc-GFP cells were grown in Eagle’s Minimum Essential Medium (EMEM, ATCC) with 10% FBS and 1% penicillin/streptomycin. Cells were incubated at 37 °C with 5% CO_2_. All transduced cell lines were compared with their WT counterpart in terms of growth and appearance, and no differences were observed.

### 2.2. GL261 Models and U87MG Model

All experiments involving animals were performed in accordance with Belgian law concerning the protection and welfare of animals and were approved by the UCLouvain ethics committee (Agreement reference: 2019/UCL/MD/004). Six-week-old female C57BL/6J mice (Charles River Laboratories, Wilmington, MA, USA) were implanted with 130,000 GL261, GL261-luc, or GL261-luc-GFP cells into the right hemisphere as previously described [20]. The maximal passage number for in vivo experiments was 10 for GL261 and GL261-luc-GFP and 20 for GL261-luc. Briefly, mice were anesthetized by intraperitoneal injection of ketamine/xylazine (100 and 13 mg/kg, respectively) and fixed in a stereotaxic frame. A burr hole was drilled using a surgical high-speed drill (Velleman, Gavere, Belgium), and cells were injected using an infusion syringe pump (Harvard Apparatus, Holliston, MA, USA) mounted with a Hamilton syringe (26S gauge needle). The injection coordinates were 2.1 mm lateral (L), 0.5 mm posterior (P) from the bregma, and 2.6 mm deep (D) from the outer border of the cranium for GL261 and GL261-luc experiments (n = 12 mice). The injection coordinates were 2.1 mm L, 0.5 mm P from the bregma, and 2.6 mm D from the outer border of the cranium (n = 6 mice), or 2.5 mm L, 0.5 mm P from the bregma, and 3.0 mm D from the outer border (n = 6 mice) for the GL261-luc-GFP experiments. Six-week-old female NMRI nude mice (Janvier, Le Genest-Saint-Isle, France) were implanted with 40,000 U87MG-Luc-GFP cells into the right striatum using the same methodology as for GL261 cells. The injection coordinates were 2.1 mm L, 0.5 mm P from the bregma, and 2.5 mm D from the outer border of the cranium.

### 2.3. Tumor Imaging

#### 2.3.1. Bioluminescence Imaging

Intracranial tumors were monitored by bioluminescence imaging using an IVIS Spectrum in vivo Imaging System (PerkinElmer, Waltham, MA, USA). Mice were anesthetized using inhaled isoflurane. Mice were injected with D-luciferin (PerkinElmer; 150 mg/kg, IP) and imaged after 15 min. The optimal time point for imaging was defined thanks to a preliminary kinetics experiment. Results are expressed as radiance (p/s).

#### 2.3.2. Magnetic Resonance Imaging

For MRI imaging, mice were anesthetized using isoflurane (3% in air for induction at 0.8 L/min and 2% in air at 0.8 L/min for maintenance). Images were obtained on a Bruker Biospec 11.7T (Germany, Ettlingen) using a birdcage volume coil (20 mm inner diameter). Animal temperature was monitored and maintained during acquisition using a rectal probe and a water heating system (warm blanket). Two imaging sequences were used. First, a flash sequence was used to obtain three anatomical images in all directions. According to the first set of images, a RARE sequence in the axial direction was acquired (TR: 2500 ms, TE: 30 ms, Rare factor: 8, number of repetitions: 8, Matrix size: 200 × 200, FOV: 20 × 20 mm^2^, slice thickness: 0.3 mm, total acquisition time: 5 min 20 s). Tumor volume measurement was done with ImageJ software. Manual delineation of tumor area was performed on every slice to assess the tumor volume. To calculate the center of mass of the tumor barycenter from each slice (coordinates *x_i_* and *y_i_*), slice position (*z_i_*) and the number of voxels (*nb_i_*) from each slice were taken into account. The position of the center of mass was estimated with this formula:

$$\bar{x}=\frac{1}{\sum_{i}{nb}_{i}}\sum_{i}x_{i}*{nb}_{i};\bar{y}=\frac{1}{\sum_{i}{nb}_{i}}\sum_{i}y_{i}*{nb}_{i;}\bar{z}=\frac{1}{\sum_{i}{nb}_{i}}\sum_{i}z_{i}*{nb}_{i}$$

The distance from the top was considered to be the difference between *z* and the top of the head.

For the acquisitions using the contrast agent, tumors were imaged using a T1 fast low-angle shot (FLASH) sequence (repetition time = 260 ms; effective echo time = 3 ms; flip angle = 25; field of view = 20 mm × 20 mm; matrix size = 200 mm × 200 mm; resolution = 10 µm; slice thickness = 0.4 mm; acquisition time = 3 min 30 s). Gadolinium (Gd)-DOTA (Dotarem^®^ 0.5 mol/mL; Guerbet, Villepinte, France) was injected intraperitoneally at a dose of 4 mmol/kg, 10 min before the acquisition.

### 2.4. In vitro Assessment of Luciferase Activity

To evaluate the luciferase activity of GL261-luc and GL261-luc-GFP cells in vitro, the ONE-Glo™ luciferase assay system (Promega, Madison, WI, USA) was used according to the manufacturer’s instructions. Briefly, 20,000 cells in 100 µL of medium were mixed with 100 µL of the kit reagent in a white 96-well plate (VWR International, Radnor, PA, USA). After 5 min, the luminescence was measured with a SpectraMax ID5 microplate reader (Molecular Devices, San Jose, CA, USA).

### 2.5. Statistical Analyses

Statistical analyses were performed with GraphPad Software Version 9.1.2. Correlations were assessed using simple linear regression. Survival studies were analyzed with the log-rank Mantel-Cox test and plotted as Kaplan-Meier curves. *p* values < 0.05 were considered significant.

## 3. Results

**Figure 1:**
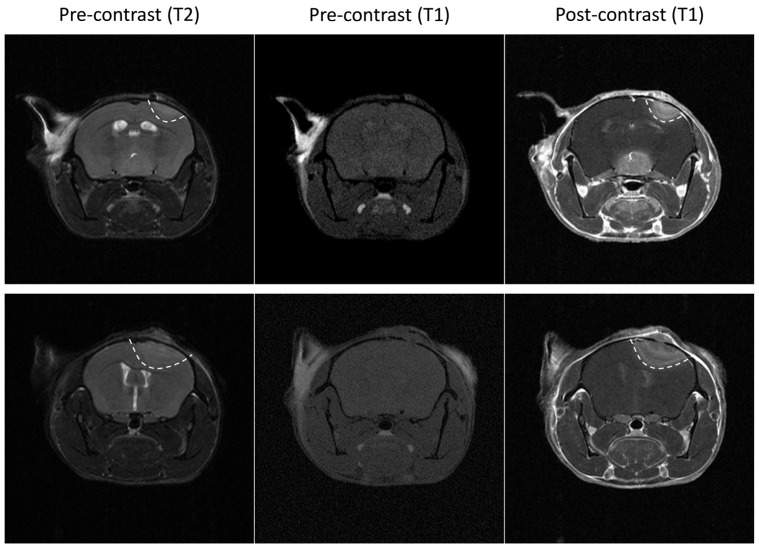
Illustrative magnetic resonance images of two wild-type GL261 tumors. Left: T2 weighted images. Note that tumors are not well delineated. Middle: pre-contrast enhanced (gadolinium (Gd)-DOTA) T1 weighted images. Note that tumors are not visible. Right: post-contrast enhanced (Gd-DOTA) T1 weighted images. Note that tumors are clearly visible, suggesting that the blood-brain barrier is disrupted in this tumor model.

After orthotopic implantation of the different GL261 cells, tumor growth was monitored using BLI with supplemental MRI analysis at several time points. The GL261 model is infiltrative into the normal brain, as previously described [21]. When small, these GL261 tumors are difficult to detect in T2-weighted MR images, and the use of contrast-enhanced T1-weighted MRI after Gd complex injection may help with their characterization. As illustrated in Figure 1, a hyperintense signal was observed in GL261 tumors after administration of Gd-DOTA compared to the pre-contrast images.

**Figure 2:**
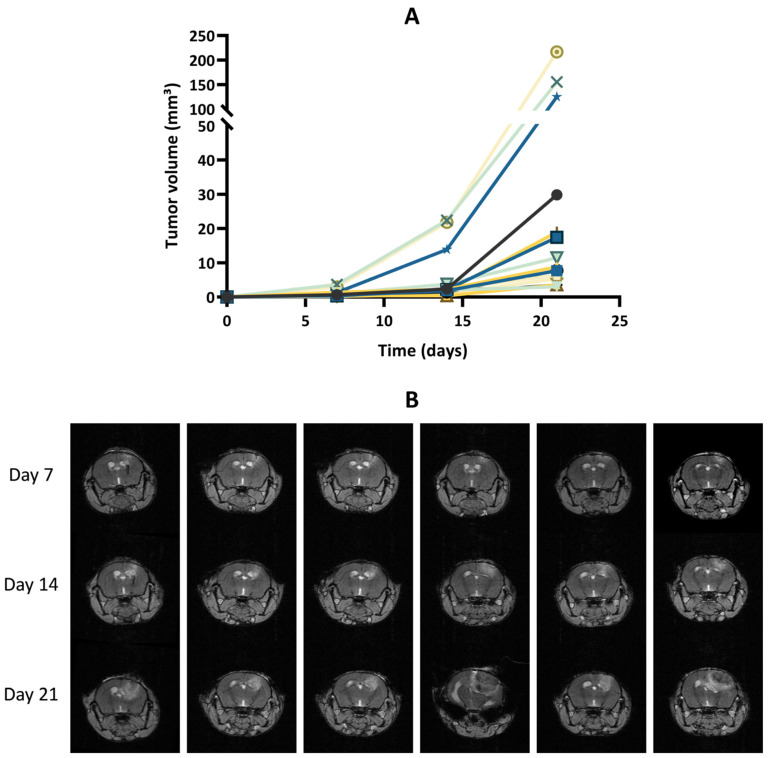
Tumor growth of GL261 tumors that do not express luciferase. (**A**) Tumor volumes measured by magnetic resonance imaging (MRI). The graph gathers the results of two in vivo experiments (N = 2) with a total of 15 mice (n = 15). (**B**) Representative T1-weighted images following gadolinium-DOTA administration. Each row represents the longitudinal measurement of tumor growth over time for an individual mouse.

**Figure 3:**
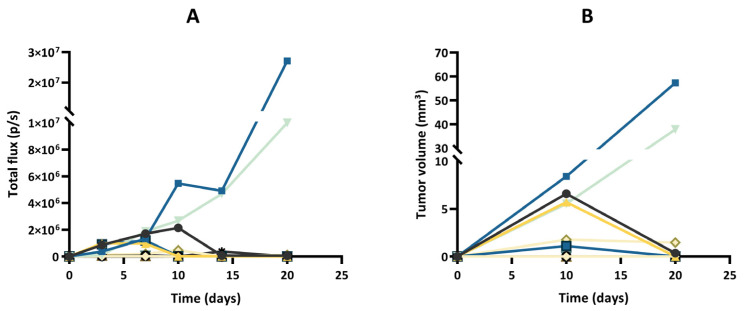
Tumor growth of the GL261-luc model, evaluated by bioluminescence imaging (BLI) and magnetic resonance imaging (MRI). (**A**) Evolution of total flux measured by BLI. (**B**) Evolution of tumor volumes measured by MRI. Each color represents one individual mouse, and the same color is used for the same mouse in both graphs; n = 12. Note that a few tumors grew rapidly, while most tumors started their growth before regressing and disappearing. Note that the results of tumor growth are consistent whatever the imaging modality used, MRI or BLI.

**Figure 4:**
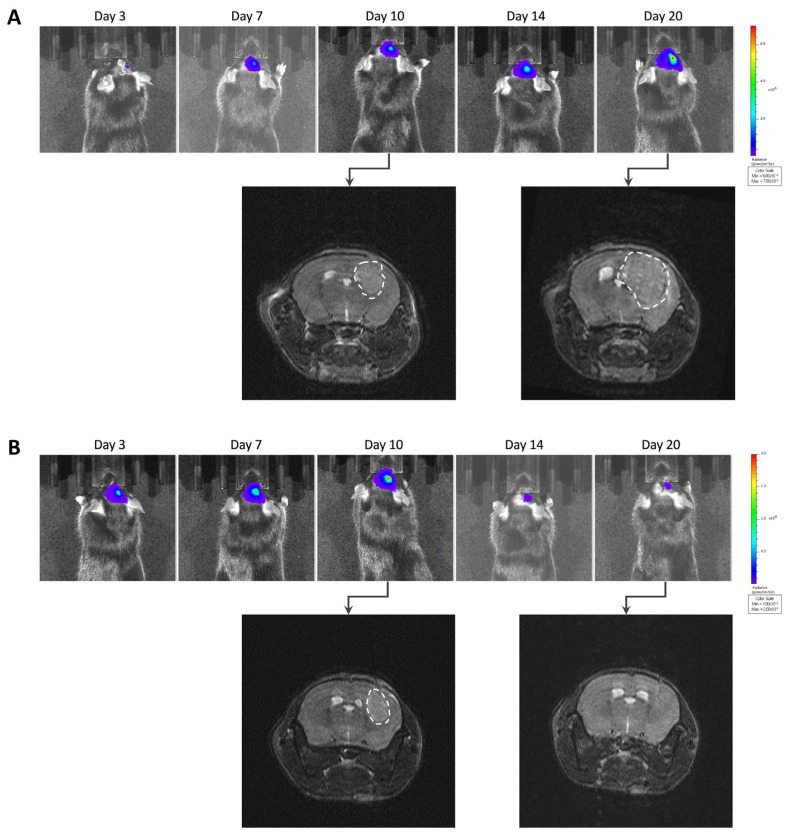
Illustrative images of GL261-luc tumor growth by bioluminescence imaging (BLI) and magnetic resonance imaging (MRI). (**A**) Representative images of a tumor growing continuously over time. (**B**) Representative images of a tumor that regressed after initial growth. Note that the results of tumor growth are consistent for this GL261 tumor model whatever the imaging modality used, MRI or BLI.

After implantation of wild-type GL261 cells, which were not expressing luciferase, into the brain parenchyma, all tumors grew over time with no sign of regression, as shown in Figure 2. In addition, the survival time of the GL261 model was reproducible between different experiments (Supplementary Figure S2). However, in our experiments on the luciferase-expressing GL261 models, tumor growth was more erratic than in the GL261 model. When using GL261-luc cells, we found that a few tumors grew rapidly, while most tumors started their growth before regressing and disappearing (Figure 3). Interestingly, for this GL261-luc model, tumor growth was consistent whatever the imaging modality used, BLI (Figure 3A) or MRI (Figure 3B). Illustrative images obtained of the GL261-luc model are presented in Figure 4. It is worth mentioning that consistent BLI and MRI tumor growth data were also obtained using the U87MG-luc-GFP glioblastoma model (Supplementary Figure S3).

**Figure 5:**
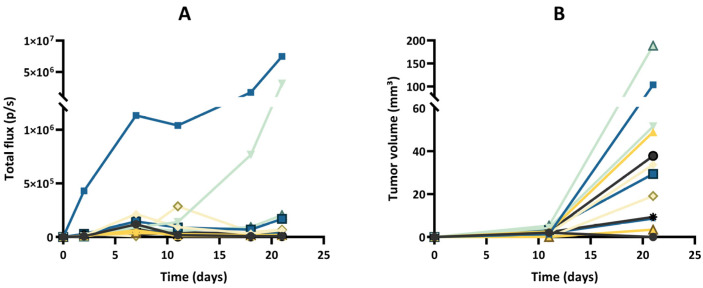
Tumor growth of the GL261-luc-GFP model, evaluated by bioluminescence imaging (BLI) and magnetic resonance imaging (MRI). (**A**) Evolution of total flux measured by BLI. (**B**) Evolution of tumor volumes measured by MRI. Each color represents one individual mouse, and the same color is used for the same mouse in both graphs; n = 12. Note that BLI suggested different growth patterns, with rapid growth for a few tumors and regression for most tumors, while MRI revealed that the tumors were present in all cases, even if their extension was variable between tumors.

**Figure 6:**
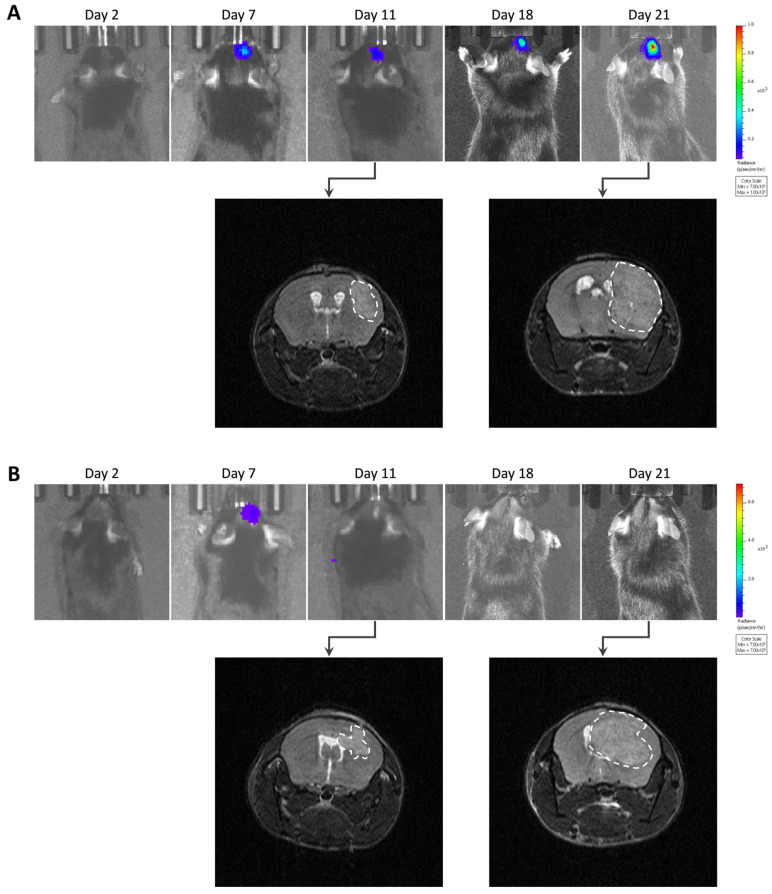
Images of GL261-luc-GFP tumor growth by bioluminescence imaging (BLI) and magnetic resonance imaging (MRI). (**A**) Representative images of a tumor’s growth with consistent data observed with both BLI and MRI. (**B**) Representative images of another case where BLI showed signal decrease over time, suggesting a regression, while MRI showed that the tumor was actually growing.

The most intriguing result regarding tumor growth was observed after inoculation of GL261 cells transfected with a specific luc-GFP construct (GL261-luc-GFP). BLI suggested different growth patterns, with rapid growth for a few tumors and regression for most tumors (Figure 5A). However, MRI revealed that the tumors were present in most cases, even if their volume was variable between tumors (Figure 5B). Illustrative images obtained for the GL261-luc-GFP model are presented in Figure 6.

**Figure 7:**
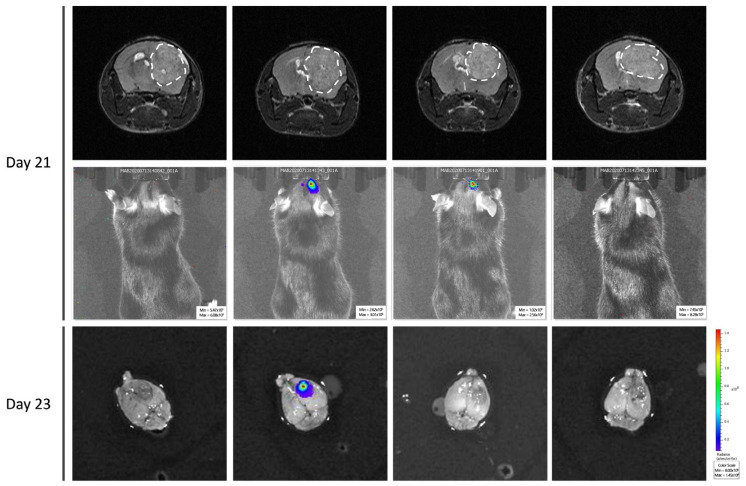
Ex vivo bioluminescence imaging to confirm the absence of light emission in GL261-luc-GFP. Four representative mice were selected. Note the discrepancy between MRI data showing large tumors in most cases, while light emission was very weak or absent in BLI.

To confirm the unexpected observations following GL261-luc-GFP implantation, we performed ex vivo BLI measurements after the sacrifice on Day 23. In Figure 7, we present illustrative results obtained in mice where MRI images are compared with BLI obtained in vivo and ex vivo just after the sacrifice. While the GBM tumors were MRI-visible in most mice, there was an absence of light emission both in vivo and ex vivo in most cases.

**Figure 8:**
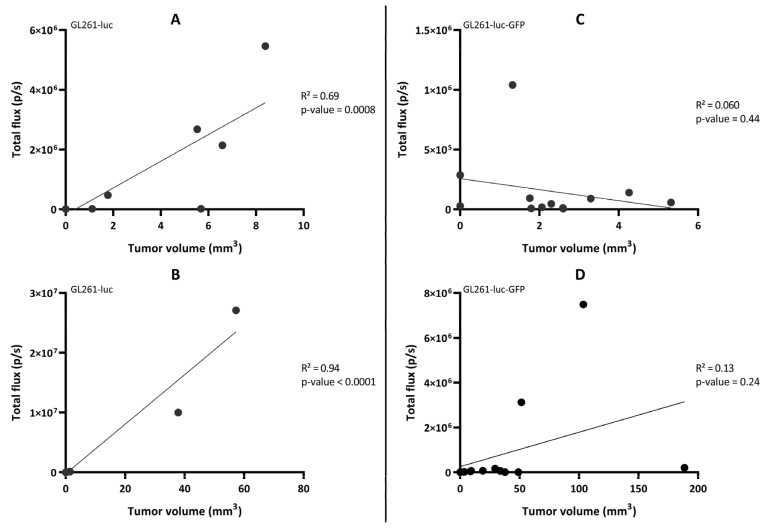
Correlation between the total flux (measured by bioluminescence imaging) and the tumor volume (measured by magnetic resonance imaging) in the GL261-luc mouse model (left panel) on Day 10 (**A**) and D20 (**B**) and the GL261-luc-GFP mouse model (right panel) on Day 11 (**C**) and Day 21 (**D**). n = 12 mice in each model. Statistical analyses were performed using simple linear regression. An enlargement of the region with low light radiance flux for each panel is presented in Figure S4.

**Figure 9:**
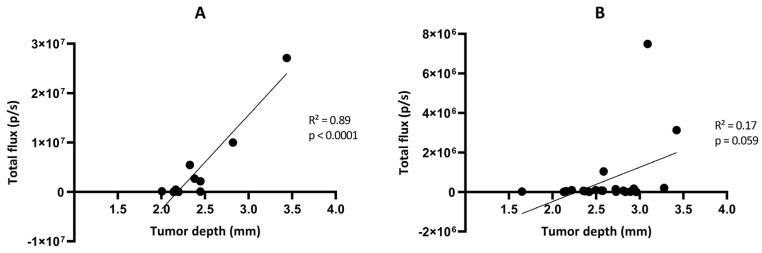
Correlation between the total flux measured by bioluminescence imaging and the tumor depth measured by magnetic resonance imaging in the GL261-luc (**A**) and GL261-luc-GFP (**B**) models. n = 10 (**A**) or 21 (**B**). Statistical analyses were performed using simple linear regression.

We further looked at the correlation between the light flux radiance and the tumor volume established by MRI for the two GBM tumor models (Figure 8). A significant correlation between light radiance and tumor volume was observed for the GL261-luc model (*p* = 0.0008; R^2^ = 0.69 on Day 10 and *p* < 0.0001; R^2^ = 0.94 on Day 20) (Figure 8A,B), while there was an absence of correlation for the GL261-luc-GFP model between light emission and tumor volume (*p* = 0.44; R^2^ = 0.060 on Day 10 and *p* = 0.24; R^2^ = 0.13 on Day 21) (Figure 8C,D). We further explored the origin of the discrepancy between both imaging modalities and investigated if the tumor could be at the origin of light attenuation, which could explain the absence of detection. For this purpose, we measured the distance between the skull surface and the barycenter of the tumor. In other words, if depth was a significant factor for the extinction of light in this orthotopic GBM model, there should have been an inverse relationship between the measured light flux radiance and the tumor depth. Whatever the model used, we did not observe such an inverse relationship between the light recorded and distance to the surface for these GBM models (Figure 9).

**Figure 10:**
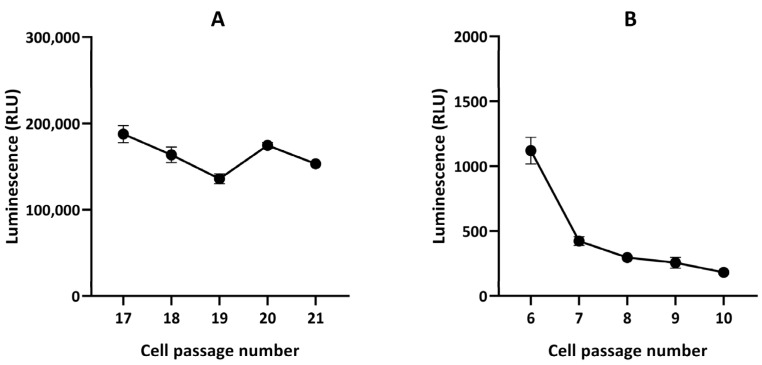
In vitro luciferase assay in GL261-luc (**A**) and GL261-luc-GFP (**B**) cells. n = 3. Note the stability of radiance flux for the GL261-luc cells and instability for the GL261-luc-GFP cells.

Finally, hypothesizing a possible instability of luciferase expression in the GL261-luc-GFP model, we investigated in vitro the stability of light emission by the cells after transfection. We observed that the emitted light significantly decreased over time in GL261-luc-GFP cells exposed to luciferin (Figure 10).

## 4. Discussion

The GL261 model is a syngeneic mouse model of GBM in C57BL/6 mice that do not require a deficient immune system [21]. The infiltrative property of GBM is a key factor for the failure of treatments [22,23], and the GL261 model is particularly suitable to represent this feature [21]. While T2-weighted MRI analysis easily depicts large GL261 tumors, the detection of tumors at an early stage can be difficult [24]. Fortunately, tumor detection can be improved by using contrast-enhanced MRI following the administration of Gd-DOTA, an extracellular paramagnetic contrast agent (Figure 1). In the clinic, this compound is administered intravenously to patients. Here, as the objective was not to gain rapid sequential images to monitor perfusion and permeability parameters [25] but rather to provide early confirmation of tumor presence as well as to assess the tumor volume, our practice has shown that the contrast can be easily obtained using an intraperitoneal injection of Gd-DOTA with images acquired 10 min after the injection.

The enhancement in MRI signal intensity following the contrast agent injection is linked to the disruption of the BBB in this tumor model. It has been reported that D-luciferin, the natural substrate of firefly luciferase, may have limited brain distribution, possibly due to the presence of BBB and efflux transporters [26,27,28,29,30]. However, as the BBB is disrupted in the GL261 model (even for small tumors), there should not be any problem with substrate availability (D-luciferin) for the luciferase expressed in the tumor cells, therefore allowing BLI studies. This is supported by the extensive use of GL261-luc cells for in vivo experiments in the literature [31,32,33,34,35,36].

One lesson learned from our study is that tumor growth was more erratic in luciferase-expressing GL261 models than in the same model that did not express luciferase. When using GL261 cells that were not expressing luciferase, all tumors grew over time with no sign of regression (Figure 2). However, in the GL261-luc model, tumor regression was observed in the majority of mice when using BLI and MRI (Figure 3 and Figure 4). Our data are consistent with recent observations that have shown that luciferase-expressing GL261 cells elicit an anti-tumor immune response by increasing pro-inflammatory modulators with a significant impact on median survival [37]. This observation is obviously crucial when trying to assess the effect of treatments by BLI. Of note, this feature has not been observed in other models (breast cancer MCF-7 and melanoma B16-F10) where high levels of luciferase did not have a negative influence on tumor cell growth [38], suggesting that the modulation of the immune response induced by luciferase expression could be model dependent.

One of the most puzzling results in our study was the discrepancy observed between BLI and MRI results when checking the evolution of the GL261-luc-GFP tumors over time (Figure 5 and Figure 6). While we observed a significant correlation between light emission and tumor volume using the G261-luc model, there was no correlation for the GL261-luc-GFP model (Figure 8). It is important to emphasize that our observations are valid for one specific construct used in the present study, and the results should not be extrapolated to all models expressing both luciferase and GFP. For example, we observed consistent results between BLI and MRI in the U87-luc-GFP model (Supplementary Figure S2). Having stated that, we tried to identify the source of the mismatch between both imaging modalities. As the tissue penetration by the luminescence signal may be affected by the localization of the tumor in tissues, as previously shown for tumors in the liver [39], we inquired if the tumor depth had any negative impact on the light radiance. However, as shown in Figure 9, this factor was not responsible for the low emission observed in the GL261-luc-GFP model.

Finally, we found that the origin of the discrepancy between BLI and MRI was the instability in luciferase expression, as we observed that the light emitted for the same number of cells decreased over time after several passages of GL261-luc-GFP cultured cells (Figure 10). Various factors affect the stability of the viral vector used. These include, but are not limited to, the specific cell transduceability, the adaptation of the specific cell to the specific promoter type, the number of promoters used in the construct (one being the best option), the type of self-cleaving peptide used (T2A, F2A, P2A, E2A, each of which is sourced from a different virus), the option of using internal ribosomal entry sites (IRIS), and much more. Our observations argue for the optimization of the transduction efficiency and stability over time [40]. The mitigation plan is first to keep checking the transduction efficiency of the cells before every experiment, and if the vector used is not optimal for the cell type used, then there are many other changes that can be made to the vector map that can help solve the problem. Ideally, the transduction efficiency of the cells should be checked before each experiment. If compromised, single-cell cloning should be performed, and cells with 90% or higher transduction efficiency should be sorted and used for in vivo experimentation. Of note, in our procedure, after the transduction, cells were sorted, and single-cell cloning was performed. After single-cell selection, 92.9% of cells were GFP-positive. Since we started noticing that the luminescence signal was lost in vivo, we decided to move forward with new GL261 cells and a Renilla vector. For the GL261-luc-GFP model used in the present study, the instability observed in luciferase expression could be due to the vector and plasmid itself or the immune reaction in vivo that may have contributed to eliminating the luciferase-positive cells.

In conclusion, the use of multi-modality imaging prevents possible errors in tumor growth evaluation, and checking the stability of luciferase expression as well as the impact of luciferase expression on tumor growth is mandatory when using BLI as the sole imaging modality.

## 5. Conclusions

The main objective of this paper was to share an experience that could have led to confusing and inappropriate interpretations of data regarding tumor growth in GBM models using BLI. We believe that researchers actively using orthotopic models of GBM could benefit from this rather negative experience that sheds light on the necessary quality control of the data.

The GL261 model is commonly used in the research of GBM immunotherapeutic treatments. Luciferase-expressing GL261 models allow the use of BLI for easy and fast longitudinal assessment of tumor growth. However, the stability of transfection as well as the impact of luciferase expression on tumor immunogenicity needs the be carefully studied while setting up such models in order to avoid misinterpretation of tumor growth.

## Data Availability

Data are contained within the article and Supplementary Materials.

## Supplementary Materials

The following supporting information can be downloaded at https://www.mdpi.com/article/10.3390/cancers15061919/s1. Supplementary method, Figure S1: Flow cytometry data analysis/FACS purification of GL261-luc-GFP cells used in the present study. Figure S2: Survival curves of untreated mice bearing GL261 tumors. Figure S3: Tumor growth of the U87MG-luc-GFP model. Figure S4: Extended Figure 8 with zoom on the region low light radiance flux.
