# Supplementary material for: Mismatch between Bioluminescence Imaging (BLI) and MRI When Evaluating Glioblastoma Growth: Lessons from a Study Where BLI Suggested “Regression” while MRI Showed “Progression”
Source: Cancers (Basel). 2023 Mar 22;15(6):1919. doi: 10.3390/cancers15061919 (PMC10047859; doi:10.3390/cancers15061919)
Supplement: Supplementary file 1 [file cancers-15-01919-s001.zip › cancers-2086675-supplementary.pdf]

Mismatch between bioluminescence imaging (BLI) and MRI when evaluating glioblastoma growth. Lessons from a study where BLI suggested "regression" while MRI showed "progression"

Mathilde Bausart [1,#], Elia Bozzato [1,#], Nicolas Joudiou [2], Xanthippi Koutsoumpou [3], Bella Manshian [3], Véronique Préat [1,*,†] and Bernard Gallez [4,*,†].

Supplementary Material

Supplementary Methods

Viral vector production

Viral vectors were engineered and produced by the Leuven Viral Vector Core (LVVC) as previously described [1]. Briefly, HIV-based SIN (self-inactivating) lentiviral vectors were produced by triple transient transfection of 293T producer cells. A VSV-G envelop encoding plasmid, a packaging plasmid together with the pCH-EF1a-eGFP-T2A-Luc2-Ires-Puro transfer plasmid were transfected using polyethylenimine (PEI; Polysciences, Amsterdam, The Netherlands). After collecting the supernatant, the medium was filtered using a 0.45 µm filter (Corning Inc., Seneffe, Belgium) and concentrated using a Vivaspin 50,000MW column (Vivascience, Bornem, Belgium). The resulting LV_EF1a-eGFP-T2A-Luc2-Ires-Puro viral vector containing concentrate was aliquoted and stored at -80 °C. After transduction, cells were treated with puromycin, sorted and single cell cloning performed. An example of single selection for the GL261-luc-GFP cells is provided in Fig. S1.

1. Ibrahimi, A.; Velde, G.V.; Reumers, V.; Toelen, J.; Thiry, I.; Vandeputte, C.; Vets, S.; Deroose, C.; Bormans, G.; Baekelandt, V.; et al. Highly Efficient Multicistronic Lentiviral Vectors with Peptide 2A Sequences. *Hum. Gene Ther.* **2009**, *20*, 845–860, doi:10.1089/hum.2008.188.

Supplementary figures

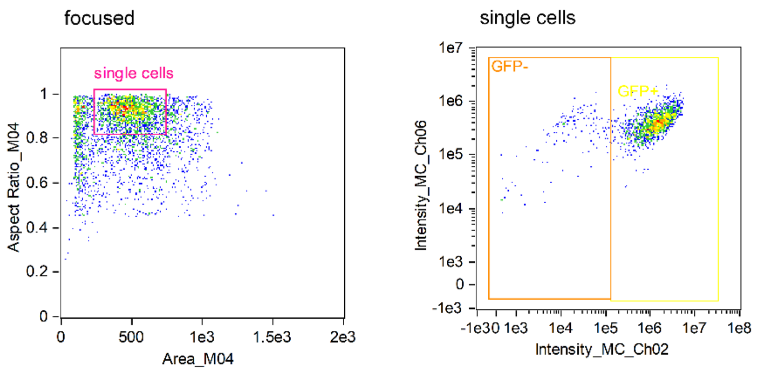

Area_M04

Population	Count	%Gated
focused	3710	100
single cells & focused	1849	49.8

Intensity_MC_Ch02

Population	Count	%Gated
single cells & focused	1849	100
GFP+ & single cells & focused	1718	92.9
GFP- & single cells & focused	131	7.08

Figure S1. Flow cytometry data analysis/FACS purification of GL261-luc-GFP cells used in the present study

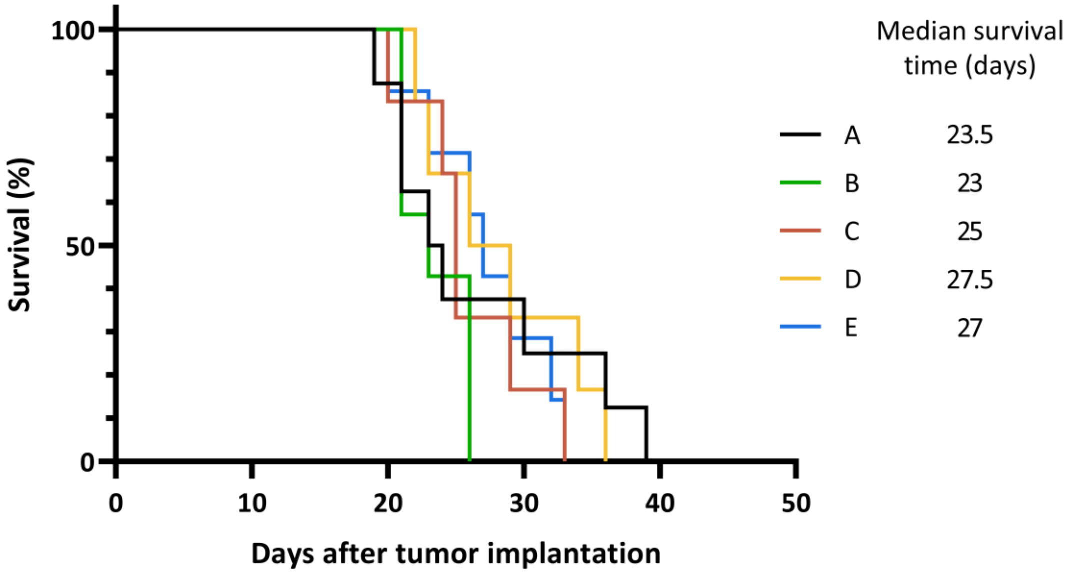

Figure S2. Survival curves of untreated mice bearing GL261 tumors. The graph gathers the results of five independent experiments (n=6-8) named from A to E. For these experiments, C57Bl/6J mice were orthotopically grafted with 1.3×10^5 GL261 cells, and then, the survival was assessed. Statistical analyses of survival curves were performed using the Mantel–Cox test and showed no difference between the curves.

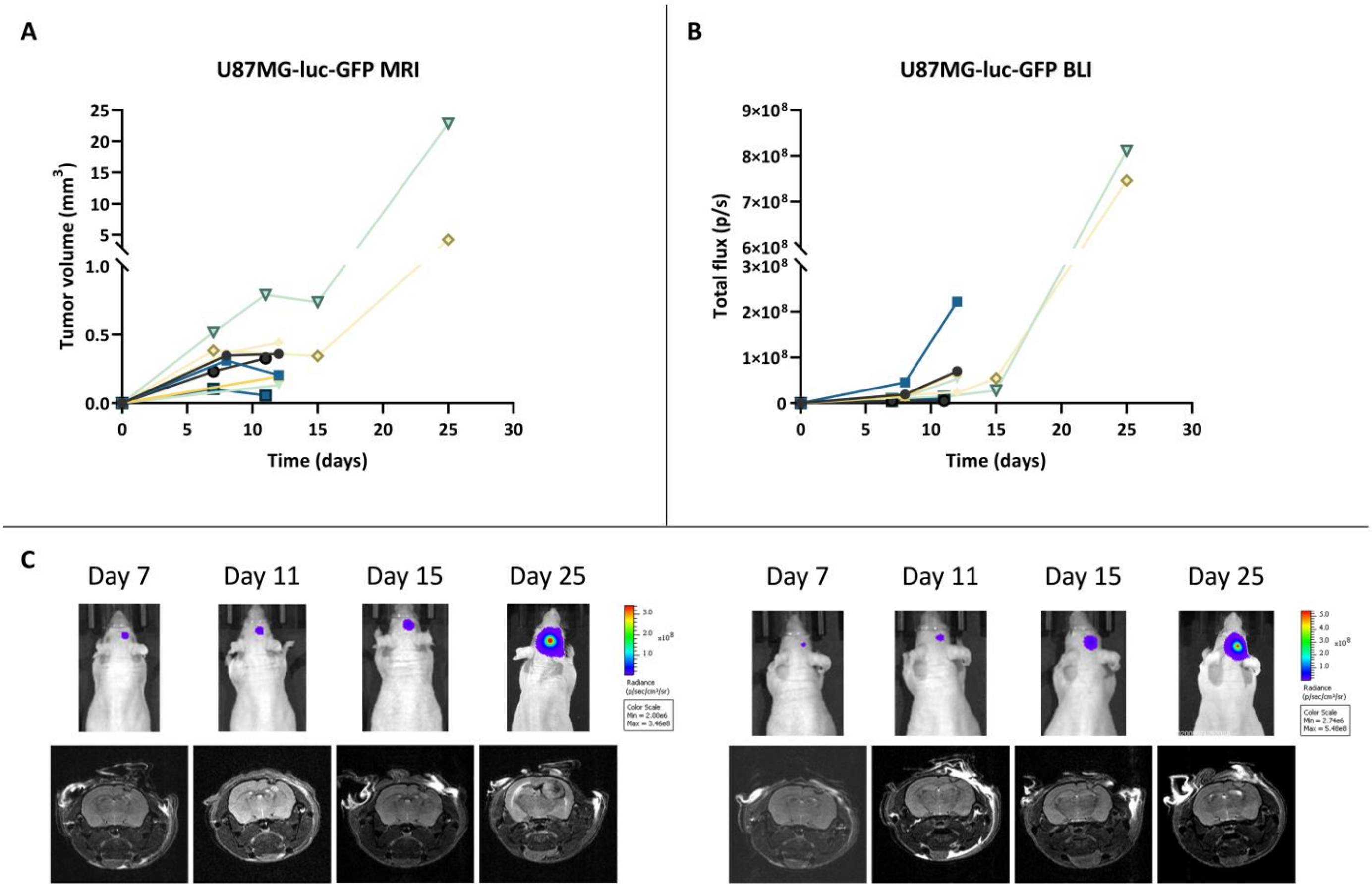

Figure S3. Tumor growth of the U87MG-luc-GFP model. (A) Evolution of tumor volumes measured by MRI (day 7-8 and day 11-12: n=9; days 15 and 25: n=2). (B) Evolution of total flux measured by BLI (day 7-8 and day 11-12: n=9; days 15 and 25: n=2). (C) Representative images of U87MG-luc-GFP tumor growth. Note that BLI and MRI results are consistent. The correlation between light flux and tumor volume defined by MRI was significant ($p < 0.0001$, R2=0.6529)

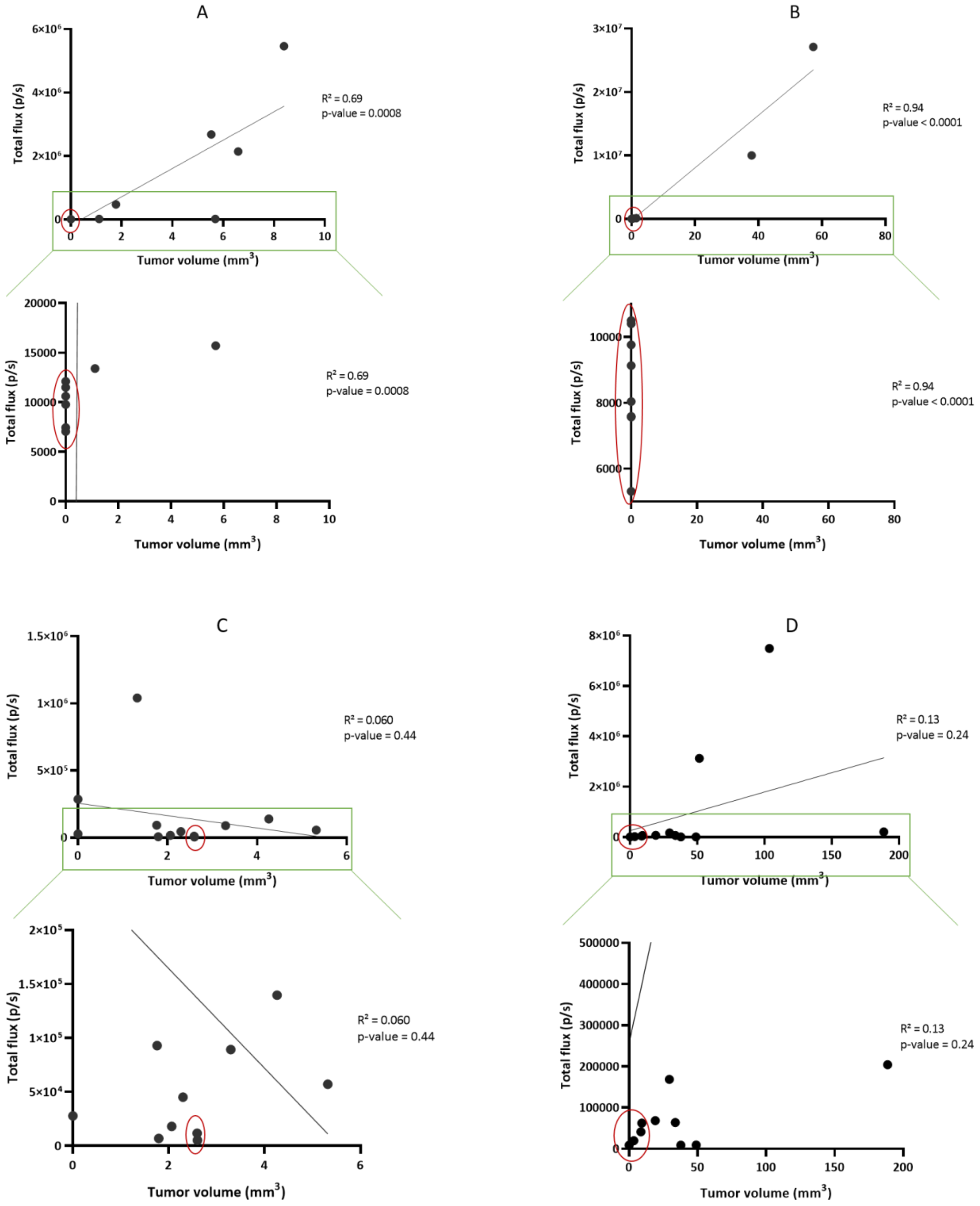

Figure S4. Extended Figure 8 with zoom on the region low light radiance flux. Correlation between the total flux (measured by bioluminescence imaging) and the tumor volume (measured by magnetic resonance imaging) in the GL261-luc mouse model (left panel) on Day 10 (A) and D20 (B) and the GL261-luc-GFP mouse model (right panel) on Day 11 (C) and Day 21 (D). n=12 mice in each model. Statistical analyses were performed using simple linear regression. An enlargement of on the region with low light radiance flux is presented for each panel,
